# Neonatal Outcomes of Infants Admitted to a Large Government Hospital in Amman, Jordan

**DOI:** 10.5539/gjhs.v7n4p217

**Published:** 2015-01-14

**Authors:** Priya G. Sivasubramaniam, Cristin E. Quinn, Meridith Blevins, Ahmand Al Hajajra, Najwa Khuri-Bulos, Samir Faouri, Natasha Halasa

**Affiliations:** 1 Vanderbilt University School of Medicine, Nashville, TN, USA; 2 Vanderbilt Institute for Global Health, 2525 West End Avenue Suite 750, Nashville, TN, 37203, USA; 3 Departments of Biostatistics, Vanderbilt University School of Medicine, Nashville, TN, USA; 4 Pediatrics, Vanderbilt University School of Medicine, Nashville, TN, USA; 5 Pediatrics, Al-Bashir Government Hospital, Amman, Jordan; 6 Pediatrics, Division of Infectious Diseases, Jordan University Hospital, Jordan

**Keywords:** Jordan, Middle East, mortality, neonatal intensive care unit (NICU), outcomes, prematurity

## Abstract

**Objective:**

To describe characteristics and outcomes of Jordanian newborns admitted to a large governmental neonatal intensive care unit (NICU).

**Methods:**

Newborns born at the government hospital, *Al Bashir*, in Amman, Jordan were prospectively enrolled. The study focused on newborns admitted to the NICU and a retrospective chart review was performed. Abstraction included in-hospital mortality, antibiotic days, ventilation, oxygen use, and CRP levels. Rank sum and chi-squared tests were used to compare across outcomes. Logistic regression of hypothesized risk factors with death adjusted for gestational age.

**Results:**

Of the 5,466 neonates enrolled from 2/10-2/11, medical records were available for 321/378(84.9%) infants admitted to the NICU. The median gestational age was 36 weeks, median birth weight was 2.3 kg, and 28(8.7%) infants died. The two most common reasons for admission and mortality were respiratory distress syndrome and prematurity. Low Apgar scores and positive CRP were predictors of mortality. Risk factors associated with increased use of antibiotics, oxygen hood, and mechanical ventilation included lower gestational age and prematurity.

**Conclusion:**

Infants admitted to the Jordanian NICU have significantly higher median gestational age and birth weights than in developed countries and were associated with significant morbidity and mortality. Continuations of global efforts to prevent prematurity are needed.

## 1. Introduction

### 1.1 Introduction

Much is known regarding outcomes of neonates admitted to the neonatal intensive care unit (NICU) in Western countries. Internationally, major causes of neonatal mortality in the NICU include birth before 37 weeks gestation (28%), infections (26%), and asphyxia (23%). In Western countries such as the United States, congenital malformations account for a significant cause of neonatal mortality (20%). Over the past three decades, global mortality rates of NICU neonates have dramatically improved due to the introduction of surfactant, steroids, and perinatal care, thereby enabling improved survival of very low birth weight infants as young as 23 to 28 weeks. As a result, neonates in countries with data regarding neonatal outcomes tend to be admitted to the NICU as young as 22 weeks for treatment, with survival rates continuing to ascend (Feng, 2011; Simpson, Xiang, Hellmann, & Tomlinson, 2010). Nonetheless, neonatal mortality continues to constitute 44% of childhood deaths under the age of five (WHO, 2013).

### 1.2 Significance

In developing countries such as those in the Middle East, data regarding NICU outcomes are sparse. Admission data, trends of mortality rates, causes of death, and NICU treatments, for instance, are unknown. While developed nations have reduced the neonatal mortality rate by 54% from 1990 to 2012, developing nations have only seen a 37% reduction in this rate, with some regions as low as 17% (WHO, 2013). Given the currently unmet United Nations Millennium Development Goal to reduce child mortality by two thirds between 1990 and 2015 and the majority of these deaths occurring in developing countries including and surrounding those of the Middle East, it is necessary report neonatal outcome data in this region (UN, 2010). To date, no comprehensive study has focused on a large, representative hospital in a Middle Eastern nation reporting general admission and outcome NICU characteristics.

### 1.3 Hypotheses and Their Correspondence to Research Design

Our group hypothesized that we would find significant differences in clinical outcomes between neonates in Western nations compared to those of a representative developing nation within the Middle East. Therefore, our group took advantage of an already existing neonatal cohort of newborns enrolled prospectively at Al-Bashir Hospital, a large government-funded hospital in Amman, Jordan, which serves as a primary hospital for the nation (Khuri-Bulos et al., 2013). The aim of our study was to determine neonatal outcome of newborns admitted to the NICU and risk factors for mortality.

## 2. Methods and/or Techniques

### 2.1 Study Design and Participants

As part of a larger study, infants born at Al Bashir hospital were prospectively enrolled at birth for a one-time assessment of their vitamin D levels (Khuri-Bulos et al., 2013). Verbal consent was obtained from mothers to obtain heel sticks for blood from their infants. A brief questionnaire assessing demographic and social behaviors of the mothers was used. All infants enrolled within 96 hours of birth were eligible for enrollment. From this cohort, neonates admitted to the NICU were identified and chart review was then performed. Al Bashir is one of three major hospitals serving Amman, Jordan. The University of Jordan, the Institutional Review Boards of Vanderbilt University, and the Jordanian Ministry of Health at Al Bashir Hospital approved this study.

### 2.2 Questionnaires

The research team used a standardized case report questionnaire. Parents were asked to provide nationality of mother and father, child’s date of birth, route of delivery, child’s birth weight, mother’s vitamin D supplementation history, daily number of hours that mother spends outdoors, mother’s clothing practice, whether or not the mother smoked during her pregnancy (and if so, which of the trimesters), and if the mother was exposed to smoke in her household during pregnancy. A database was implemented using REDCap (Research Electronic Data Capture), a secure, web-based application designed to support data capture for research studies, created and hosted at Vanderbilt University (Harris, Taylor, Thielke, Payne, Gonzalez, & Conde, 2009).

### 2.3 Clinical Outcomes Measures

Data were abstracted from the clinical charts of the neonates admitted to the NICU to collect admission and outcome measures. The major outcomes of interested included: in-hospital mortality, antibiotic days, mechanical ventilation, oxygen use, and elevated (c-reactive protein) CRP levels. In-hospital mortality referred to infant death prior to NICU discharge. Antibiotic days referred to the total number of days on all antibiotics, with information regarding specific antibiotics used. Mechanical ventilation and oxygen use as well as total number of days were calculated. Elevated CRP was defined as having a CRP above a set value at any point during the infant’s hospitalization in the NICU.

### 2.4 Statistical Analyses

Descriptive statistics were used to summarize sociodemographic and clinical characteristics for each aforementioned NICU outcome, and tests of association included Wilcoxon rank sum and chi-square tests. The dependence of duration of antibiotic use on gestational age, sepsis, RDS, birth weight, Apgar score, vitamin D, premature rupture of membranes (PROM), preeclampsia, and positive CRP was modeled using negative binomial regression; only babies who were alive at discharge were included due to truncation by death. Due to a limited number of deaths, 11 different logistic regression models were estimated for a set of 11 covariates while adjusting for gestational age to prevent over-fitting a larger model. To account for possible non-linear associations, continuous variables were included using restricted cubic splines. Model covariates were identified *a priori* and no adjustment was made for multiple comparisons. We employed R-software 2.15.1 (www.r-project.org) for all data analyses. Analysis scripts are available at http://biostat.mc.vanderbilt.edu/ArchivedAnalyses.

## 3. Results

### 3.1 Patient Population Characteristics and Recruitment

From February 2010 to July 2011, 378 out of 5,466 neonates in our prospective cohort were admitted to the NICU. Of these 378 infants, medical records were available for 321 (85%). Median gestational age of neonates admitted to the NICU was 36 weeks (interquartile range [IQR] 34-37 weeks), median birth weight was 2.3 kg (IQR 1.9-2.9 kg), and 154 (48%) of these neonates were female. Median length of stay in the NICU was 4 days (range 2-8 days) and the two most common reasons for admission were respiratory distress syndrome (67%) and prematurity (52%). Table 1 provides a summary of patient population characteristics.

**Table 1:** NICU Characteristics by Vital Status at Discharge, Oxyhood, and Ventilator Use Vital Status

	Dead (n = 28)	Alive (n = 293)	Combined (n = 321)	P-value
Female	14 (50%)	140 (48%)	154 (48%)	0.98
Gestational age	30 (29 - 33)	36 (35 - 37)	36 (34 - 37)	<0.001
Missing, n(%)	0 (0%)	4 (1%)	4 (1%)	
Days in hospital	6 (2 - 11)	4 (3 - 8)	4 (2 - 8)	0.65
Reason(s) for admission				
Sepsis	6 (21%)	25 (9%)	31 (10%)	0.061
RDS	24 (86%)	191 (65%)	215 (67%)	0.046
Neonatal pneumonia	1 (4%)	0 (0%)	1 (<1%)	0.14
Prematurity	21 (75%)	146 (50%)	167 (52%)	0.019
Heart Disease	2 (7%)	4 (1%)	6 (2%)	0.15
Congenital Malformation	4 (14%)	14 (5%)	18 (6%)	0.097
Overweight Infant	0 (0%)	17 (6%)	17 (5%)	0.39
Low birth weight infant	3 (11%)	42 (14%)	45 (14%)	0.81
Jaundice	0 (0%)	16 (5%)	16 (5%)	0.42
Low Apgar score	4 (14%)	15 (5%)	19 (6%)	0.12
Seizures	0 (0%)	1 (<1%)	1 (<1%)	0.99
Hypoglycemia	0 (0%)	4 (1%)	4 (1%)	0.99
Neonatal Asphyxia	3 (11%)	11 (4%)	14 (4%)	0.22
Other	5 (18%)	94 (32%)	99 (31%)	0.18
Birth weight (kg)	1.2 (0.9 - 1.9)	2.3 (2 - 2.9)	2.3 (1.9 - 2.9)	<0.001
Missing, n(%)	0 (0%)	1 (<1%)	1 (<1%)	
Weight at 5 days (kg)	1.3 (1.2 - 1.6)	2.07 (1.785 - 2.65)	2 (1.73 - 2.6)	<0.001
Missing, n(%)	19 (68%)	194 (66%)	213 (66%)	
Apgar at 1 minute	4 (3 - 5)	7 (6 - 7)	7 (5 - 7)	<0.001
Missing, n(%)	7 (25%)	135 (46%)	142 (44%)	
Apgar at 5 minutes	6 (5 - 7.5)	8 (7 - 8)	8 (7 - 8)	<0.001
Missing, n(%)	5 (18%)	113 (39%)	118 (37%)	
CRP (ever positive), n(%)				<0.001
Missing	9 (32%)	21 (7%)	30 (9%)	
Negative	12 (63%)	255 (94%)	267 (92%)	
Positive	7 (37%)	17 (6%)	24 (8%)	
Received surfactant	11 (39%)	15 (5%)	26 (8%)	<0.001
Days on antibiotics	6 (2 - 10)	4 (2 - 7)	4 (2 - 7)	0.44
WBC count (1st value)	15.4 (9.7 - 25.4)	13.2 (9.9 - 17.1)	13.3 (9.9 - 17.3)	0.21
Missing, n(%)	2 (7%)	6 (2%)	8 (2%)	
Vent	24 (86%)	6 (2%)	30 (9%)	<0.001
Vent days	2.5 (1 - 6.25)	3 (2 - 5.5)	2.5 (1 - 6)	
Nasal canula	20 (71%)	98 (33%)	118 (37%)	<0.001
Nasal canula days	2 (1.5 - 4.5)	1 (1 - 2)	1 (1 - 2)	
Oxyhood	9 (32%)	184 (63%)	193 (60%)	0.003
Oxyhood days	6 (1 - 8)	1 (1 - 2)	1 (1 - 2)	
Incubator	2 (7%)	107 (37%)	109 (34%)	0.003
Incubator days	6 (3 - 8)	1 (1 - 2)	1 (1 - 2)	
Delivery, n(%)				0.61
Missing	1 (4%)	2 (1%)	3 (1%)	
Vaginal	12 (44%)	150 (52%)	162 (51%)	
Cesarean	15 (56%)	141 (48%)	156 (49%)	
Gestational DM	1 (4%)	21 (7%)	22 (7%)	0.74
PROM	3 (11%)	19 (6%)	22 (7%)	0.65
Preeclampsia	5 (18%)	26 (9%)	31 (10%)	0.23

Oxyhood use				

	**Oxyhood (n=193)**	**No Oxyhood (n=128)**	**Combined (n=321)**	**P-value**

Female	91 (47%)	63 (49%)	154 (48%)	0.80
Gestational age	35 (34 - 37)	37 (35 - 37)	36 (34 - 37)	0.002
Missing, n(%)	0 (0%)	4 (3%)	4 (1%)	
Days in hospital	5 (3 - 9)	3 (2 - 6)	4 (2 - 8)	<0.001
Reason(s) for admission				
Sepsis	14 (7%)	17 (13%)	31 (10%)	0.11
RDS	159 (82%)	56 (44%)	215 (67%)	<0.001
Neonatal pneumonia	0 (0%)	1 (1%)	1 (<1%)	0.84
Prematurity	118 (61%)	49 (38%)	167 (52%)	<0.001
Heart Disease	2 (1%)	4 (3%)	6 (2%)	0.35
Congenital Malformation	11 (6%)	7 (5%)	18 (6%)	0.99
Overweight Infant	5 (3%)	12 (9%)	17 (5%)	0.016
Low birth weight infant	27 (14%)	18 (14%)	45 (14%)	0.99
Jaundice	8 (4%)	8 (6%)	16 (5%)	0.56
Low Apgar score	12 (6%)	7 (5%)	19 (6%)	0.97
Seizures	0 (0%)	1 (1%)	1 (<1%)	0.84
Hypoglycemia	1 (1%)	3 (2%)	4 (1%)	0.35
Neonatal Asphyxia	8 (4%)	6 (5%)	14 (4%)	0.99
Other	44 (23%)	55 (43%)	99 (31%)	<0.001
Death	9 (5%)	19 (15%)	28 (9%)	
Birth weight (kg)	2.2 (1.9 - 2.8)	2.5 (2.1 - 3.1)	2.29 (1.9 - 2.9)	0.005
Missing, n(%)	1 (1%)	0 (0%)	1 (<1%)	
Weight at 5 days (kg)	1.9 (1.8 - 2.5)	2.36 (1.665 - 2.95)	2 (1.7 - 2.6)	0.32
Missing, n(%)	112 (58%)	101 (79%)	213 (66%)	
Apgar at 1 minute	6 (5 - 7)	7 (5 - 7)	7 (5 - 7)	0.80
Missing, n(%)	79 (41%)	63 (49%)	142 (44%)	
Apgar at 5 minutes	8 (7 - 8)	8 (6 - 8)	8 (7 - 8)	0.39
Missing, n(%)	66 (34%)	52 (41%)	118 (37%)	
Vitamin D	3.5 (2.5 - 4.8)	3.5 (2.5 - 5.3)	3.5 (2.5 - 5)	0.63
Missing, n(%)	70 (36%)	37 (29%)	107 (33%)	
CRP (ever positive), n(%)				0.82
Missing	11 (6%)	19 (15%)	30 (9%)	
Negative	168 (92%)	99 (91%)	267 (92%)	
Positive	14 (8%)	10 (9%)	24 (8%)	
Received surfactant	17 (9%)	9 (7%)	26 (8%)	0.717
Days on antibiotics	4 (3 - 8)	3 (2 - 5)	4 (2 - 7)	<0.001
WBC count (1st value)	12.3 (9.7 - 16.5)	15 (10.5 - 18.9)	13.3 (9.9 - 17.3)	0.003
Missing, n(%)	4 (2%)	4 (3%)	8 (2%)	
Vent	11 (6%)	19 (15%)	30 (9%)	0.010
Vent days	3 (1.5 - 5)	2 (1 - 6.5)	2.5 (1 - 6)	
Nasal canula	67 (35%)	51 (40%)	118 (37%)	0.42
Nasal canula days	1 (1 - 2.8)	1 (1 - 2)	1 (1 - 2)	
Incubator	84 (44%)	25 (20%)	109 (34%)	<0.001
Incubator days	2 (1 - 2)	1 (1 - 2)	1 (1 - 2)	
Delivery, n(%)				0.99
Missing	2 (1%)	1 (1%)	3 (1%)	
Vaginal	97 (51%)	65 (51%)	162 (51%)	
Cesarean	94 (49%)	62 (49%)	156 (49%)	
Gestational DM	9 (5%)	13 (10%)	22 (7%)	0.093
PROM	11 (6%)	11 (9%)	22 (7%)	0.44
Preeclampsia	19 (10%)	12 (9%)	31 (10%)	0.99

Mechanical Ventilation				

	**Ventilator (n=30)**	**No Ventilator (n=291)**	**Combined (n=321)**	**P-value**

Female	17 (57%)	137 (47%)	154 (48%)	0.42
Gestational age	31 (29.3 - 354.8)	36 (35 - 37)	36 (34 - 37)	<0.001
Missing, n(%)	0 (0%)	4 (1%)	4 (1%)	
Days in hospital	109.5 (3 - 14.8)	4 (2 - 8)	4 (2 - 8)	0.012
Reason(s) for admission				
Sepsis	6 (20%)	25 (9%)	31 (10%)	0.091
RDS	25 (83%)	190 (65%)	215 (67%)	0.072
Neonatal pneumonia	1 (3%)	0 (0%)	1 (<1%)	0.16
Prematurity	21 (70%)	146 (50%)	167 (52%)	0.060
Heart Disease	3 (10%)	3 (1%)	6 (2%)	0.006
Congenital Malformation	3 (10%)	15 (5%)	18 (6%)	0.50
Overweight Infant	0 (0%)	17 (6%)	17 (5%)	0.35
Low birth weight infant	4 (13%)	41 (14%)	45 (14%)	0.99
Jaundice	0 (0%)	16 (5%)	16 (5%)	0.38
Low Apgar score	5 (17%)	14 (5%)	19 (6%)	0.027
Seizures	0 (0%)	1 (<1%)	1 (<1%)	0.99
Hypoglycemia	0 (0%)	4 (1%)	4 (1%)	0.99
Neonatal Asphyxia	4 (13%)	10 (3%)	14 (4%)	0.040
Other	7 (23%)	92 (32%)	99 (31%)	0.47
Death	24 (80%)	4 (1%)	28 (9%)	
Birth weight (kg)	1.55 (1 - 2)	2.3 (2 - 2.9)	2.29 (1.9 - 2.9)	<0.001
Missing, n(%)	0 (0%)	1 (<1%)	1 (<1%)	
Weight at 5 days (kg)	1.6 (1.2 - 1.9)	2.07 (1.77 - 2.63)	2 (1.73 - 2.6)	0.006
Missing, n(%)	17 (57%)	196 (67%)	213 (66%)	
Apgar at 1 minute	4 (2.75 - 5)	7 (6 - 7)	7 (5 - 7)	<0.001
Missing, n(%)	6 (20%)	136 (47%)	142 (44%)	
Apgar at 5 minutes	6 (5 - 7)	8 (7 - 8)	8 (7 - 8)	<0.001
Missing, n(%)	5 (17%)	113 (39%)	118 (37%)	
Vitamin D	3.7 (2.985 - 5.765)	3.5 (2.5 - 5)	3.5 (2.5 - 5)	0.48
Missing, n(%)	11 (37%)	96 (33%)	107 (33%)	
CRP (ever positive), n(%)				<0.001
Missing	7 (23%)	23 (8%)	30 (9%)	
Negative	13 (57%)	254 (95%)	267 (92%)	
Positive	10 (43%)	14 (5%)	24 (8%)	
Received surfactant	10 (33%)	16 (5%)	26 (8%)	<0.001
Days on antibiotics	9 (2 - 14)	4 (2 - 7)	4 (2 - 7)	0.006
WBC count (1st value)	15.4 (10.2 - 26.4)	13.2 (9.9 - 17)	13.3 (9.9 - 17.3)	0.11
Missing, n(%)	2 (7%)	6 (2%)	8 (2%)	
Nasal canula	24 (80%)	94 (32%)	118 (37%)	<0.001
Nasal canula days	2 (1 - 5)	1 (1 - 2)	1 (1 - 2)	
Oxyhood	11 (37%)	182 (63%)	193 (60%)	0.010
Oxyhood days	6 (1.5 - 8)	1 (1 - 2)	1 (1 - 2)	
Incubator	6 (20%)	103 (35%)	109 (34%)	0.14
Incubator days	3 (2.25 - 3)	1 (1 - 2)	1 (1 - 2)	
Delivery, n(%)				0.38
Missing	1 (3%)	2 (1%)	3 (1%)	
Vaginal	12 (41%)	150 (52%)	162 (51%)	
Cesarean	17 (59%)	139 (48%)	156 (49%)	
Gestational DM	1 (3%)	21 (7%)	22 (7%)	0.67
PROM	2 (7%)	20 (7%)	22 (7%)	0.99
Preeclampsia	6 (20%)	25 (9%)	31 (10%)	0.091

### 3.2 Antibiotic Usage

Excluding neonates who died during their stay, we characterized neonates based upon their duration of antibiotic treatment (1-7 days vs. >7 days, Table 2).

**Table 2:** NICU characteristics by extended antibiotic use (excluding deaths)

	>7 Days (n=61)	1-7 Days (n=227)	Combined (n=288)	P-value
Female, n(%)	31 (51%)	104 (46%)	135 (48%)	0.58
Gestational age, median (IQR)	34 (32 - 36)	37 (35 - 37)	36 (35 - 37)	<0.001
Missing, n(%)	1 (2%)	3 (1%)	4 (1%)	
Days in hospital, median (IQR)	14 (10 - 24)	3 (2 - 5)	4 (3 - 8)	<0.001
Reason(s) for admission^2^, n(%)				
Sepsis	10 (16%)	15 (7%)	25 (9%)	0.031
RDS	51 (84%)	137 (60%)	188 (65%)	0.001
Prematurity	44 (72%)	101 (44%)	145 (50%)	<0.001
Heart Disease	1 (2%)	2 (1%)	3 (1%)	0.99
Congenital Malformation	4 (7%)	10 (4%)	14 (5%)	0.72
Overweight Infant	0 (0%)	17 (7%)	17 (6%)	0.058
Low birth weight infant	11 (18%)	29 (13%)	40 (14%)	0.40
Jaundice	5 (8%)	11 (5%)	16 (5%)	0.48
Low Apgar score	6 (10%)	8 (4%)	14 (5%)	0.089
Seizures	0 (0%)	1 (<1%)	1 (<1%)	0.99
Hypoglycemia	0 (0%)	4 (2%)	4 (1%)	0.67
Neonatal Asphyxia	5 (8%)	5 (2%)	10 (4%)	0.061
Other	16 (26%)	77 (34%)	93 (32%)	0.32
Birth weight (kg)	1.8325 (1.7 - 2.213)	2.5 (2.15 - 3)	2.3 (2 - 2.9)	<0.001
Missing, n(%)	1 (2%)	0 (0%)	1 (<1%)	
Weight at 5 days (kg)	1.8 (1.6 - 2.3)	2.4 (2.0 - 2.9)	2.1 (1.8 - 2.7)	<0.001
Missing, n(%)	13 (21%)	176 (78%)	189 (66%)	
Apgar at 1 minute	6 (4.3 - 7)	7 (6 - 7)	7 (6 - 7)	0.001
Missing, n(%)	19 (31%)	115 (51%)	134 (47%)	
Apgar at 5 minutes	8 (6 - 8)	8 (7.75 - 8)	8 (7 - 8)	0.007
Missing, n(%)	17 (28%)	95 (42%)	112 (39%)	
Vitamin D	3.655 (2.3 - 5.475)	3.5 (2.6 - 5)	3.5 (2.5 - 5)	0.83
Missing, n(%)	21 (34%)	75 (33%)	96 (33%)	
CRP (ever positive), n(%)				0.086
Missing	3 (5%)	17 (7%)	20 (7%)	
Negative	51 (88%)	200 (95%)	251 (94%)	
Positive	7 (12%)	10 (5%)	17 (6%)	
Received surfactant	11 (18%)	4 (2%)	15 (5%)	<0.001
WBC count (1st value)	10.8 (8.4 - 16.8)	13.3 (10.3 - 17.1)	13.2 (9.9 - 17.1)	0.046
Missing, n(%)	0 (0%)	6 (3%)	6 (2%)	
Vent	5 (8%)	0 (0%)	5 (2%)	<0.001
Vent days	4 (2 - 6)	-	3 (2 - 5.5)	
Nasal canula	39 (64%)	56 (25%)	95 (33%)	<0.001
Nasal canula days	1 (1 - 43.75)	1 (1 - 1)	1 (1 - 2)	
Oxyhood	51 (84%)	130 (57%)	181 (63%)	<0.001
Oxyhood days	2 (1 - 3.25)	1 (1 - 1)	1 (1 - 2)	
Incubator	34 (56%)	71 (31%)	105 (37%)	<0.001
Incubator days	2 (1 - 3)	1 (1 - 2)	1 (1 - 2)	
Delivery, n(%)				0.035
Missing	0 (0%)	2 (1%)	2 (1%)	
Vaginal	24 (39%)	125 (56%)	149 (52%)	
Cesarean	37 (61%)	100 (44%)	137 (48%)	
Gestational DM	3 (5%)	18 (8%)	21 (7%)	0.60
PROM	6 (10%)	13 (6%)	19 (6%)	0.39
Preeclampsia	9 (15%)	16 (7%)	25 (9%)	0.10

Percentages are computed using the number of infants with a non-missing value. IQR: interquartile range

^1^ To compare the distribution of patient characteristics by antibiotic use, we employ chi-square tests. Similarly, we use Wilcoxon rank sum tests for continuous variables by antibiotic use.

2 Percentages may not sum to 100%.

Factors that had a significant effect upon antibiotic use included lower gestational age, respiratory distress syndrome, prematurity, lower weight at birth and at five days, use of surfactant, and all methods of respiratory support (p<0.001). Negative binomial regression was utilized to determine independent predictors of length of antibiotic use (Table 3).

**Table 3:** Model Effects: Duration of Antibiotic Use

	Incidence Rate Ratio (95% CI)	P-value
Gestational age (per 1 week)	0.98 (0.93, 1.03)	0.48
Admitted for sepsis	1.46 (1.09, 1.95)	0.010
Admitted for RDS	1.12 (0.91, 1.37)	0.28
Birth weight (3 vs. 2 kg)	0.57 (0.48, 0.67)	<0.001
Apgar at 1 minute (per 2 increase)	0.80 (0.70, 0.91)	<0.001
Vitamin D (per 2.6 increase)	1.09 (0.99, 1.21)	0.067
PROM	0.99 (0.71, 1.39)	0.97
Preeclampsia	1.30 (0.99, 1.72)	0.058
Positive CRP	1.48 (1.02, 2.15)	0.040

Admission for sepsis and positive CRP increase the expected number of days on antibiotics by some 46% and 48%, respectively. Birthweight of 3 kg compared with 2 kg is associated with a 43% decrease in days on antibiotics (Incidence Rate Ratio [IRR]: 0.57; 95% confidence interval [CI]: 0.48-0.67). A two-point increase in Apgar score at one minute is associated with a 20% reduction in antibiotic days (IRR: 0.80; 95% CI: 0.70-0.91). We failed to detect any association between gestational age, respiratory distress syndrome admission, vitamin D, PROM and preeclampsia with antibiotic use.

### 3.3 Oxygen and Mechanical Ventilation

Neonates who utilized oxygen compared to those who did not had a longer median hospital stay (5 vs. 3 days, p < 0.001), more admissions for respiratory distress syndrome (82% vs. 44%, p < 0.001), more prematurity (61% vs. 38%, p < 0.001), more days on antibiotics (4 vs. 3 days, p < 0.001), and were more likely to be placed in an incubator (44% vs. 20%, p < 0.001) (Table 1).

Neonates who utilized mechanical ventilation compared to those who did not had a lower median gestational age (31 vs. 36 weeks, p < 0.001), lower median birth weight (1.6 vs. 2.3 kg, p < 0.001), lower Apgar scores at one and five minutes (4 and 6 vs. 7 and 8, p < 0.001), more likely to receive surfactant (33% vs. 5%, p < 0.001), and more likely to have been placed nasal canula (80% vs. 32%, p < 0.001) (Table 1).

### 3.4 Mortality

Of 321 total infants admitted to the NICU, 28 infants (8.7%) died prior to discharge. The most common admission diagnoses in infants who died were respiratory distress syndrome (86%, p = 0.046) and prematurity (75%, p = 0.019). Comparing neonates who died in the NICU to those who survived, neonates who died had a lower gestational age (30 weeks vs. 36 weeks, p < 0.001); lower median birth weight (1.2 kg vs. 2.3 kg, p < 0.001); and lower Apgar scores at one and five minutes (4 and 6 vs. 7 and 8, p < 0.001). Infants who died were more likely to have received surfactant than those who survived (39% vs. 5%, p < 0.001), require mechanical ventilation 86% vs. 2%, p < 0.001) and more likely to have been placed on nasal canula (71% v. 33%, p < 0.001) (Table 1).

Adjusting for gestational age in separate logistic regression models, Apgar scores, use of mechanical ventilation or oxygen, and an elevated CRP level were associated with death at discharge. A two-point increase in Apgar at 1 minute was associated with a 74% decrease in the odds of death (Odds Ratio [OR]: 0.26; 95% CI: 0.12-0.57). A one-point increase in Apgar at 5 minutes was associated with a 44% decrease in the odds of death (OR: 0.56; 95% CI: 0.37-0.83). Elevated CRP level was associated with a 10.5 time higher odds of death (OR: 10.52; 95% CI: 2.25-49.2). No significant associations were detected for gender, sepsis, respiratory distress syndrome, nasal canula, birth weight, maternal preeclampsia, method of delivery, and PROM with neonatal mortality (Table 4).

**Table 4:** Model Effects: Odds of Death at Discharge (adjusted for gestational age)

	N	Odds Ratio^1^ (95% CI)	P-value
Female	317	1.80 (0.63, 5.14)	0.27
Admitted for sepsis	317	1.10 (0.24, 4.97)	0.90
Admitted for RDS	317	0.66 (0.15, 2.84)	0.57
Birth weight (3 vs. 2 kg)	316	0.44 (0.17, 1.09)	0.11
Apgar at 1 minute (per 2 increase)	178	0.26 (0.12, 0.57)	<0.001
Apgar at 5 minutes (per 1 increase)	201	0.56 (0.37, 0.83)	0.004
Days on antibiotics (per 5 days)	312	0.73 (0.48, 1.13)	0.16
PROM	308	0.69 (0.10, 4.71)	0.71
Preeclampsia	308	2.41 (0.61, 9.48)	0.21
Positive CRP	288	10.52 (2.25, 49.16)	0.003
C-section (vs. vaginal)	314	0.51 (0.17, 1.57)	0.24
Received mechanical ventalation	316	776.00 (57.50, 10473.00)	<0.001
Received oxyhood	314	0.26 (0.08, 0.78)	0.017
Received nasal canula	314	2.40 (0.74, 7.80)	0.15

1 Adjusted for gestational age at birth.

## 4. Discussion

In a prospective cohort study at the Al-Bashir NICU in Amman, Jordan, the most common reasons for admission were respiratory distress syndrome and prematurity, and their median gestational age would be considered late pre-term infants (>36 weeks). Our cohort had an overall mortality of 8.7% and those neonates who died had a median gestational age of 30 weeks. Neonatal morbidity has grown to represent a larger proportion of overall infant mortality during the last two decades in Jordan, with prematurity as the leading cause of the mortality rate of children under five years of age (34%), followed by congenital anomalies (21%) and birth asphyxia (12%) (Country, 2008-2013; WHO, 2013; Khoury & Mas’ad, 2002). This is compatible with global data, with 40% of deaths in children younger than five years occurring in the neonatal period, and 99% of neonatal deaths occurring in low- or middle-income countries (Lawn, Wilczynska-Ketenda, & Cousens, 2006).

The 2010 World Health Organization (WHO) data regarding neonatal mortality indicate that 989 of 1,975 (50%) neonates aged 0 to 27 days are due to complications from prematurity. Our study is consistent with these findings, as half of the neonates admitted to the NICU were premature with a higher percentage of premature neonates requiring oxygen. Moreover, neonates that required mechanical ventilation or died had even lower gestational ages than those who did not require mechanical ventilation or those who survived. Since nearly 15% of all childhood deaths under age five are caused by prematurity-related complications, medical management of prematurity constitutes a large part of reducing childhood mortality and must be emphasized in developing nations such as Jordan with high rates of prematurity (WHO, 2013).

Current data pertaining to NICU outcomes in the United States has established that neonates admitted with gestational ages of 26 weeks have mortality rates as low as 9.7% (Sparks, Caughey, & Cheng, 2014). Infants with gestational ages of 22 to 26 weeks are often viable and can be considered for admission to the NICU (Sparks et al., 2014). However, although the median gestational age of infants admitted to this NICU in Jordan is 36 weeks, it is not always beneficial for resource-poor countries to admit extremely premature infants (< 28 weeks). Extreme prematurity is associated with morbidities including retinopathy, patent ductus arteriosus, bronchopulmonary dysplasia, respiratory syncytial virus infection, interventricular hemorrhage, developmental delay, and necrotizing enterocolitis, which is a heavy financial burden for these resource-limited countries (Shapiro-Mendoza et al., 2008; Chang, 2011). In a Canadian study, cost per infant during the first ten years of life for gestational ages less than 28 weeks was $67,467 versus $10,010 for infants born at 33-36 weeks due to such complications (Johnston, Gooch, Korol, Vo, Eyawo, Bradt, & Levy 2014). In the United States, cost for term infants is around $2,061 versus $26,054 for late-preterm infants, and costs within the first year of life were three times higher for late-preterm infants (McLaurin, Hall, Jackson, Owens, & Mahadevia, 2009). While cost analyses have not been conducted in Jordan, it is difficult to appropriate funds in resource-limited nations to sufficiently treat certain severe prematurity-associated complications (Johnston et al., 2014; Abu-Salah, 2011). However, other prematurity-associated complications such as retinopathy of prematurity can be screened for in the neonatal setting within a developing nation and treated early in a cost-effective manner (Zin, Magluta, Pinto, Entringer, Mendes-Gomes, Moreira, & Gilbert, 2014). Furthermore, some complications such as low birth weight can potentially be prevented with improved prenatal maternal follow-up and education (Dai, Mao, Luo, & Shen, 2014). Therefore, in order to diminish neonatal mortality in such nations, organizations such as the WHO must consider financial aspects of treatment.

Despite the high financial burden of caring for extremely premature infants in a developing nation, there are preliminary steps that can be taken to decrease current costs of neonatal healthcare. One aspect of improvement includes the current lack of universal screening for group B Streptococcus (GBS) in Jordan. As a result, GBS status in mothers is unknown and likely contributes to a significant percentage of the cases of sepsis and respiratory distress syndrome from this cohort. Provision of routine screening for infections such as GBS and CMV along with prophylactic treatment would be a cost-effective method to diminish subsequent costs of treatment and extended hospital stays (Alkhawaja, Ismaeel, Botta, & Senok, 2012). A second area of improvement of neonatal health in this study includes respiratory distress syndrome (RDS), which was a key exposure for mortality, extended antibiotic use, and requiring oxygen. This high rate of RDS could be due to preventable environmental exposures such as the high rates of smoking, including Amman (Khuri-Bulos et al., 2013). Environmental exposures could also be a reason for the low median birth weight found in this cohort (2.3 kg), which was a significant factor for extended antibiotic use, mechanical ventilation, and mortality.

This study is the first to provide comprehensive information regarding outcomes of neonates in a representative cohort in the Middle Eastern region. Although this study is the first to describe neonatal outcomes in Amman, Jordan, there are certain limitations. Chart abstraction was acquired through paper records, with missing records, which could potentially alter statistics such as mortality rate and life-threatening prematurity-related complications such as interventricular hemorrhage. Furthermore, due to suboptimal microbiological conditions, not all neonates had documented infections. Our results indicate that efforts must be made in order to improve treatment of neonates currently admitted to the NICU and prevent neonatal morbidities such as RDS and deaths that we know to be avoidable per data from developed nations. Pertinent variables include maternal prenatal and perinatal care, and medication and equipment used in the NICU. Also, we must determine preventable environmental causes of neonatal respiratory distress syndrome and prematurity and also evaluate how to fund excess costs incurred by the treatment of extremely premature infants. These efforts will help meet the United Nations’ goal of a reduction of neonatal mortality. Of note, neonatal medicine has been introduced to Middle Eastern nations within the past two decades, while neonatal medicine was established in the United States by 1973. Therefore, the current discrepancies in neonatal care could also be attributed to the relative novelty of this field of care and resources in nations such as Jordan (Rezaeizadeh, Nayeri, & Shariat, 2014).

## 5. Conclusion

In a retrospective analysis of Middle Eastern NICU outcomes, infants admitted to the Jordanian NICU have significantly higher median gestational age and birth weights than in developed countries. Among this population, low Apgar scores, use of mechanical ventilation or oxygen, and positive CRP were associated with mortality. Further data collection is necessary in order to increase the power of this study and follow-up studies are necessary in order to work towards the current United Nations Millennium Development Goal of reducing childhood mortality. Longitudinal studies are needed to characterize subsequent outcomes that are not measured in this study.

## Appendix A

**List of Tables Included in Manuscript**

**Table 1A:** NICU Characteristics by Vital Status at Discharge, Oxyhood, and Ventilator Use Vital Status

	Dead (n = 28)	Alive (n = 293)	Combined (n = 321)	P-value
Female	14 (50%)	140 (48%)	154 (48%)	0.98
Gestational age	30 (29 - 33)	36 (35 - 37)	36 (34 - 37)	<0.001
Missing, n(%)	0 (0%)	4 (1%)	4 (1%)	
Days in hospital	6 (2 - 11)	4 (3 - 8)	4 (2 - 8)	0.65
Reason(s) for admission				
Sepsis	6 (21%)	25 (9%)	31 (10%)	0.061
RDS	24 (86%)	191 (65%)	215 (67%)	0.046
Neonatal pneumonia	1 (4%)	0 (0%)	1 (<1%)	0.14
Prematurity	21 (75%)	146 (50%)	167 (52%)	0.019
Heart Disease	2 (7%)	4 (1%)	6 (2%)	0.15
Congenital Malformation	4 (14%)	14 (5%)	18 (6%)	0.097
Overweight Infant	0 (0%)	17 (6%)	17 (5%)	0.39
Low birth weight infant	3 (11%)	42 (14%)	45 (14%)	0.81
Jaundice	0 (0%)	16 (5%)	16 (5%)	0.42
Low Apgar score	4 (14%)	15 (5%)	19 (6%)	0.12
Seizures	0 (0%)	1 (<1%)	1 (<1%)	0.99
Hypoglycemia	0 (0%)	4 (1%)	4 (1%)	0.99
Neonatal Asphyxia	3 (11%)	11 (4%)	14 (4%)	0.22
Other	5 (18%)	94 (32%)	99 (31%)	0.18
Birth weight (kg)	1.2 (0.9 - 1.9)	2.3 (2 - 2.9)	2.3 (1.9 - 2.9)	<0.001
Missing, n(%)	0 (0%)	1 (<1%)	1 (<1%)	
Weight at 5 days (kg)	1.3 (1.2 - 1.6)	2.07 (1.785 - 2.65)	2 (1.73 - 2.6)	<0.001
Missing, n(%)	19 (68%)	194 (66%)	213 (66%)	
Apgar at 1 minute	4 (3 - 5)	7 (6 - 7)	7 (5 - 7)	<0.001
Missing, n(%)	7 (25%)	135 (46%)	142 (44%)	
Apgar at 5 minutes	6 (5 - 7.5)	8 (7 - 8)	8 (7 - 8)	<0.001
Missing, n(%)	5 (18%)	113 (39%)	118 (37%)	
CRP (ever positive), n(%)				<0.001
Missing	9 (32%)	21 (7%)	30 (9%)	
Negative	12 (63%)	255 (94%)	267 (92%)	
Positive	7 (37%)	17 (6%)	24 (8%)	
Received surfactant	11 (39%)	15 (5%)	26 (8%)	<0.001
Days on antibiotics	6 (2 - 10)	4 (2 - 7)	4 (2 - 7)	0.44
WBC count (1st value)	15.4 (9.7 - 25.4)	13.2 (9.9 - 17.1)	13.3 (9.9 - 17.3)	0.21
Missing, n(%)	2 (7%)	6 (2%)	8 (2%)	
Vent	24 (86%)	6 (2%)	30 (9%)	<0.001
Vent days	2.5 (1 - 6.25)	3 (2 - 5.5)	2.5 (1 - 6)	
Nasal canula	20 (71%)	98 (33%)	118 (37%)	<0.001
Nasal canula days	2 (1.5 - 4.5)	1 (1 - 2)	1 (1 - 2)	
Oxyhood	9 (32%)	184 (63%)	193 (60%)	0.003
Oxyhood days	6 (1 - 8)	1 (1 - 2)	1 (1 - 2)	
Incubator	2 (7%)	107 (37%)	109 (34%)	0.003
Incubator days	6 (3 - 8)	1 (1 - 2)	1 (1 - 2)	
Delivery, n(%)				0.61
Missing	1 (4%)	2 (1%)	3 (1%)	
Vaginal	12 (44%)	150 (52%)	162 (51%)	
Cesarean	15 (56%)	141 (48%)	156 (49%)	
Gestational DM	1 (4%)	21 (7%)	22 (7%)	0.74
PROM	3 (11%)	19 (6%)	22 (7%)	0.65
Preeclampsia	5 (18%)	26 (9%)	31 (10%)	0.23

Oxyhood use

	**Oxyhood (n=193)**	**No Oxyhood (n=128)**	**Combined (n=321)**	**P-value**

Female	91 (47%)	63 (49%)	154 (48%)	0.80
Gestational age	35 (34 - 37)	37 (35 - 37)	36 (34 - 37)	0.002
Missing, n(%)	0 (0%)	4 (3%)	4 (1%)	
Days in hospital	5 (3 - 9)	3 (2 - 6)	4 (2 - 8)	<0.001
Reason(s) for admission				
Sepsis	14 (7%)	17 (13%)	31 (10%)	0.11
RDS	159 (82%)	56 (44%)	215 (67%)	<0.001
Neonatal pneumonia	0 (0%)	1 (1%)	1 (<1%)	0.84
Prematurity	118 (61%)	49 (38%)	167 (52%)	<0.001
Heart Disease	2 (1%)	4 (3%)	6 (2%)	0.35
Congenital Malformation	11 (6%)	7 (5%)	18 (6%)	0.99
Overweight Infant	5 (3%)	12 (9%)	17 (5%)	0.016
Low birth weight infant	27 (14%)	18 (14%)	45 (14%)	0.99
Jaundice	8 (4%)	8 (6%)	16 (5%)	0.56
Low Apgar score	12 (6%)	7 (5%)	19 (6%)	0.97
Seizures	0 (0%)	1 (1%)	1 (<1%)	0.84
Hypoglycemia	1 (1%)	3 (2%)	4 (1%)	0.35
Neonatal Asphyxia	8 (4%)	6 (5%)	14 (4%)	0.99
Other	44 (23%)	55 (43%)	99 (31%)	<0.001
Death	9 (5%)	19 (15%)	28 (9%)	
Birth weight (kg)	2.2 (1.9 - 2.8)	2.5 (2.1 - 3.1)	2.29 (1.9 - 2.9)	0.005
Missing, n(%)	1 (1%)	0 (0%)	1 (<1%)	
Weight at 5 days (kg)	1.9 (1.8 - 2.5)	2.36 (1.665 - 2.95)	2 (1.7 - 2.6)	0.32
Missing, n(%)	112 (58%)	101 (79%)	213 (66%)	
Apgar at 1 minute	6 (5 - 7)	7 (5 - 7)	7 (5 - 7)	0.80
Missing, n(%)	79 (41%)	63 (49%)	142 (44%)	
Apgar at 5 minutes	8 (7 - 8)	8 (6 - 8)	8 (7 - 8)	0.39
Missing, n(%)	66 (34%)	52 (41%)	118 (37%)	
Vitamin D	3.5 (2.5 - 4.8)	3.5 (2.5 - 5.3)	3.5 (2.5 - 5)	0.63
Missing, n(%)	70 (36%)	37 (29%)	107 (33%)	
CRP (ever positive), n(%)				0.82
Missing	11 (6%)	19 (15%)	30 (9%)	
Negative	168 (92%)	99 (91%)	267 (92%)	
Positive	14 (8%)	10 (9%)	24 (8%)	
Received surfactant	17 (9%)	9 (7%)	26 (8%)	0.717
Days on antibiotics	4 (3 - 8)	3 (2 - 5)	4 (2 - 7)	<0.001
WBC count (1st value)	12.3 (9.7 - 16.5)	15 (10.5 - 18.9)	13.3 (9.9 - 17.3)	0.003
Missing, n(%)	4 (2%)	4 (3%)	8 (2%)	
Vent	11 (6%)	19 (15%)	30 (9%)	0.010
Vent days	3 (1.5 - 5)	2 (1 - 6.5)	2.5 (1 - 6)	
Nasal canula	67 (35%)	51 (40%)	118 (37%)	0.42
Nasal canula days	1 (1 - 2.8)	1 (1 - 2)	1 (1 - 2)	
Incubator	84 (44%)	25 (20%)	109 (34%)	<0.001
Incubator days	2 (1 - 2)	1 (1 - 2)	1 (1 - 2)	
Delivery, n(%)				0.99
Missing	2 (1%)	1 (1%)	3 (1%)	
Vaginal	97 (51%)	65 (51%)	162 (51%)	
Cesarean	94 (49%)	62 (49%)	156 (49%)	
Gestational DM	9 (5%)	13 (10%)	22 (7%)	0.093
PROM	11 (6%)	11 (9%)	22 (7%)	0.44
Preeclampsia	19 (10%)	12 (9%)	31 (10%)	0.99

Mechanical Ventilation

	**Ventilator (n=30)**	**No Ventilator (n=291)**	**Combined (n=321)**	**P-value**

Female	17 (57%)	137 (47%)	154 (48%)	0.42
Gestational age	31 (29.3 - 354.8)	36 (35 - 37)	36 (34 - 37)	<0.001
Missing, n(%)	0 (0%)	4 (1%)	4 (1%)	
Days in hospital	109.5 (3 - 14.8)	4 (2 - 8)	4 (2 - 8)	0.012
Reason(s) for admission				
Sepsis	6 (20%)	25 (9%)	31 (10%)	0.091
RDS	25 (83%)	190 (65%)	215 (67%)	0.072
Neonatal pneumonia	1 (3%)	0 (0%)	1 (<1%)	0.16
Prematurity	21 (70%)	146 (50%)	167 (52%)	0.060
Heart Disease	3 (10%)	3 (1%)	6 (2%)	0.006
Congenital Malformation	3 (10%)	15 (5%)	18 (6%)	0.50
Overweight Infant	0 (0%)	17 (6%)	17 (5%)	0.35
Low birth weight infant	4 (13%)	41 (14%)	45 (14%)	0.99
Jaundice	0 (0%)	16 (5%)	16 (5%)	0.38
Low Apgar score	5 (17%)	14 (5%)	19 (6%)	0.027
Seizures	0 (0%)	1 (<1%)	1 (<1%)	0.99
Hypoglycemia	0 (0%)	4 (1%)	4 (1%)	0.99
Neonatal Asphyxia	4 (13%)	10 (3%)	14 (4%)	0.040
Other	7 (23%)	92 (32%)	99 (31%)	0.47
Death	24 (80%)	4 (1%)	28 (9%)	
Birth weight (kg)	1.55 (1 - 2)	2.3 (2 - 2.9)	2.29 (1.9 - 2.9)	<0.001
Missing, n(%)	0 (0%)	1 (<1%)	1 (<1%)	
Weight at 5 days (kg)	1.6 (1.2 - 1.9)	2.07 (1.77 - 2.63)	2 (1.73 - 2.6)	0.006
Missing, n(%)	17 (57%)	196 (67%)	213 (66%)	
Apgar at 1 minute	4 (2.75 - 5)	7 (6 - 7)	7 (5 - 7)	<0.001
Missing, n(%)	6 (20%)	136 (47%)	142 (44%)	
Apgar at 5 minutes	6 (5 - 7)	8 (7 - 8)	8 (7 - 8)	<0.001
Missing, n(%)	5 (17%)	113 (39%)	118 (37%)	
Vitamin D	3.7 (2.985 - 5.765)	3.5 (2.5 - 5)	3.5 (2.5 - 5)	0.48
Missing, n(%)	11 (37%)	96 (33%)	107 (33%)	
CRP (ever positive), n(%)				<0.001
Missing	7 (23%)	23 (8%)	30 (9%)	
Negative	13 (57%)	254 (95%)	267 (92%)	
Positive	10 (43%)	14 (5%)	24 (8%)	
Received surfactant	10 (33%)	16 (5%)	26 (8%)	<0.001
Days on antibiotics	9 (2 - 14)	4 (2 - 7)	4 (2 - 7)	0.006
WBC count (1st value)	15.4 (10.2 - 26.4)	13.2 (9.9 - 17)	13.3 (9.9 - 17.3)	0.11
Missing, n(%)	2 (7%)	6 (2%)	8 (2%)	
Nasal canula	24 (80%)	94 (32%)	118 (37%)	<0.001
Nasal canula days	2 (1 - 5)	1 (1 - 2)	1 (1 - 2)	
Oxyhood	11 (37%)	182 (63%)	193 (60%)	0.010
Oxyhood days	6 (1.5 - 8)	1 (1 - 2)	1 (1 - 2)	
Incubator	6 (20%)	103 (35%)	109 (34%)	0.14
Incubator days	3 (2.25 - 3)	1 (1 - 2)	1 (1 - 2)	
Delivery, n(%)				0.38
Missing	1 (3%)	2 (1%)	3 (1%)	
Vaginal	12 (41%)	150 (52%)	162 (51%)	
Cesarean	17 (59%)	139 (48%)	156 (49%)	
Gestational DM	1 (3%)	21 (7%)	22 (7%)	0.67
PROM	2 (7%)	20 (7%)	22 (7%)	0.99
Preeclampsia	6 (20%)	25 (9%)	31 (10%)	0.091

**Table 1B:** NICU characteristics by extended antibiotic use (excluding deaths)

	>7 Days (n=61)	1-7 Days (n=227)	Combined (n=288)	P-value
Female, n(%)	31 (51%)	104 (46%)	135 (48%)	0.58
Gestational age, median (IQR)	34 (32 - 36)	37 (35 - 37)	36 (35 - 37)	<0.001
Missing, n(%)	1 (2%)	3 (1%)	4 (1%)	
Days in hospital, median (IQR)	14 (10 - 24)	3 (2 - 5)	4 (3 - 8)	<0.001
Reason(s) for admission^2^, n(%)				
Sepsis	10 (16%)	15 (7%)	25 (9%)	0.031
RDS	51 (84%)	137 (60%)	188 (65%)	0.001
Prematurity	44 (72%)	101 (44%)	145 (50%)	<0.001
Heart Disease	1 (2%)	2 (1%)	3 (1%)	0.99
Congenital Malformation	4 (7%)	10 (4%)	14 (5%)	0.72
Overweight Infant	0 (0%)	17 (7%)	17 (6%)	0.058
Low birth weight infant	11 (18%)	29 (13%)	40 (14%)	0.40
Jaundice	5 (8%)	11 (5%)	16 (5%)	0.48
Low Apgar score	6 (10%)	8 (4%)	14 (5%)	0.089
Seizures	0 (0%)	1 (<1%)	1 (<1%)	0.99
Hypoglycemia	0 (0%)	4 (2%)	4 (1%)	0.67
Neonatal Asphyxia	5 (8%)	5 (2%)	10 (4%)	0.061
Other	16 (26%)	77 (34%)	93 (32%)	0.32
Birth weight (kg)	1.8325 (1.7 - 2.213)	2.5 (2.15 - 3)	2.3 (2 - 2.9)	<0.001
Missing, n(%)	1 (2%)	0 (0%)	1 (<1%)	
Weight at 5 days (kg)	1.8 (1.6 - 2.3)	2.4 (2.0 - 2.9)	2.1 (1.8 - 2.7)	<0.001
Missing, n(%)	13 (21%)	176 (78%)	189 (66%)	
Apgar at 1 minute	6 (4.3 - 7)	7 (6 - 7)	7 (6 - 7)	0.001
Missing, n(%)	19 (31%)	115 (51%)	134 (47%)	
Apgar at 5 minutes	8 (6 - 8)	8 (7.75 - 8)	8 (7 - 8)	0.007
Missing, n(%)	17 (28%)	95 (42%)	112 (39%)	
Vitamin D	3.655 (2.3 - 5.475)	3.5 (2.6 - 5)	3.5 (2.5 - 5)	0.83
Missing, n(%)	21 (34%)	75 (33%)	96 (33%)	
CRP (ever positive), n(%)				0.086
Missing	3 (5%)	17 (7%)	20 (7%)	
Negative	51 (88%)	200 (95%)	251 (94%)	
Positive	7 (12%)	10 (5%)	17 (6%)	
Received surfactant	11 (18%)	4 (2%)	15 (5%)	<0.001
WBC count (1st value)	10.8 (8.4 - 16.8)	13.3 (10.3 - 17.1)	13.2 (9.9 - 17.1)	0.046
Missing, n(%)	0 (0%)	6 (3%)	6 (2%)	
Vent	5 (8%)	0 (0%)	5 (2%)	<0.001
Vent days	4 (2 - 6)	-	3 (2 - 5.5)	
Nasal canula	39 (64%)	56 (25%)	95 (33%)	<0.001
Nasal canula days	1 (1 - 43.75)	1 (1 - 1)	1 (1 - 2)	
Oxyhood	51 (84%)	130 (57%)	181 (63%)	<0.001
Oxyhood days	2 (1 - 3.25)	1 (1 - 1)	1 (1 - 2)	
Incubator	34 (56%)	71 (31%)	105 (37%)	<0.001
Incubator days	2 (1 - 3)	1 (1 - 2)	1 (1 - 2)	
Delivery, n(%)				0.035
Missing	0 (0%)	2 (1%)	2 (1%)	
Vaginal	24 (39%)	125 (56%)	149 (52%)	
Cesarean	37 (61%)	100 (44%)	137 (48%)	
Gestational DM	3 (5%)	18 (8%)	21 (7%)	0.60
PROM	6 (10%)	13 (6%)	19 (6%)	0.39
Preeclampsia	9 (15%)	16 (7%)	25 (9%)	0.10

Percentages are computed using the number of infants with a non-missing value. IQR: interquartile range ^1^ To compare the distribution of patient characteristics by antibiotic use, we employ chi-square tests. Similarly, we use Wilcoxon rank sum tests for continuous variables by antibiotic use.

2 Percentages may not sum to 100%.

**Table 1C:** Model Effects: Duration of Antibiotic Use

	Incidence Rate Ratio (95% CI)	P-value
Gestational age (per 1 week)	0.98 (0.93, 1.03)	0.48
Admitted for sepsis	1.46 (1.09, 1.95)	0.010
Admitted for RDS	1.12 (0.91, 1.37)	0.28
Birth weight (3 vs. 2 kg)	0.57 (0.48, 0.67)	<0.001
Apgar at 1 minute (per 2 increase)	0.80 (0.70, 0.91)	<0.001
Vitamin D (per 2.6 increase)	1.09 (0.99, 1.21)	0.067
PROM	0.99 (0.71, 1.39)	0.97
Preeclampsia	1.30 (0.99, 1.72)	0.058
Positive CRP	1.48 (1.02, 2.15)	0.040

**Table 1D:** Model Effects: Odds of Death at Discharge (adjusted for gestational age)

	N	Odds Ratio^1^ (95% CI)	P-value
Female	317	1.80 (0.63, 5.14)	0.27
Admitted for sepsis	317	1.10 (0.24, 4.97)	0.90
Admitted for RDS	317	0.66 (0.15, 2.84)	0.57
Birth weight (3 vs. 2 kg)	316	0.44 (0.17, 1.09)	0.11
Apgar at 1 minute (per 2 increase)	178	0.26 (0.12, 0.57)	<0.001
Apgar at 5 minutes (per 1 increase)	201	0.56 (0.37, 0.83)	0.004
Days on antibiotics (per 5 days)	312	0.73 (0.48, 1.13)	0.16
PROM	308	0.69 (0.10, 4.71)	0.71
Preeclampsia	308	2.41 (0.61, 9.48)	0.21
Positive CRP	288	10.52 (2.25, 49.16)	0.003
C-section (vs. vaginal)	314	0.51 (0.17, 1.57)	0.24
Received mechanical ventilation	316	776.00 (57.50, 10473.00)	<0.001
Received oxyhood	314	0.26 (0.08, 0.78)	0.017
Received nasal canula	314	2.40 (0.74, 7.80)	0.15

1 Adjusted for gestational age at birth.
